# Supplementary material for: The genome sequence of the Alpine Blue, Agriades orbitulus (de Prunner, 1798) (Lepidoptera: Lycaenidae)
Source: Wellcome Open Res. 2025 Jul 25;10:362. [Version 1] doi: 10.12688/wellcomeopenres.24607.1 (PMC12552840; doi:10.12688/wellcomeopenres.24607.1)
Supplement: Peer review history

# Reviewer report: Reviewer response for version 1

**DOI:** 10.21956/wellcomeopenres.27117.r134196

**Published:** 2025-10-25

**Authors:** Samridhi Chaturvedi, Garima Setia

**Affiliations:** 1 Tulane University, New Orleans, Louisiana, USA; 2 Ecology and Evolutionary Biology, Tulane University, New Orleans, Louisiana, USA

The manuscript "The genome sequence of the Alpine Blue, *Agriades orbitulus* (de Prunner, 1798) (Lepidoptera: Lycaenidae)" presents a high-quality genome assembly with good scaffold N50 and BUSCO completeness. The authors have used appropriate tools for genome assembly and sequence data analysis. The manuscript is overall well written.

 However, I propose the following minor corrections:

 • More background information about the ecology, life cycle, and behavior of the species would be helpful.

 • The introduction should explain more clearly the relevance of this species/ genome assembly.

 • Please include the versions of all programs used for the assembly and analysis to ensure reproducibility.

 • The k-mer completeness is below the expected benchmark of 95 percent. This should be addressed in the text whether the lower individual completeness is acceptable, expected, or not.

 • The manuscript should clarify why haplotype 2 was not assembled to chromosome level.

 • The section- “Sample acquisition and DNA barcoding” does not actually include any information about DNA barcoding. This part seems to have been accidentally left out and should be completed.

 • It would also be helpful to include more information explaining the figures, either in the captions or in the main text.

 This genome will be a valuable resource for future research in Lepidoptera. I recommend that the authors revise and resubmit the manuscript after making these minor changes.

Are sufficient details of methods and materials provided to allow replication by others?

Partly

Is the rationale for creating the dataset(s) clearly described?

Yes

Are the datasets clearly presented in a useable and accessible format?

Yes

Are the protocols appropriate and is the work technically sound?

Partly

Reviewer Expertise:

Evolutionary genomics, speciation, hybridization, high-throughput genome sequencing and assembly

We confirm that we have read this submission and believe that we have an appropriate level of expertise to confirm that it is of an acceptable scientific standard.

# Reviewer report: Reviewer response for version 1

**DOI:** 10.21956/wellcomeopenres.27117.r134195

**Published:** 2025-10-25

**Authors:** Nitin Ravikanthachari

**Affiliations:** 1 University of Montana, Missoula, Montana, USA

The authors report a high-quality genome for the Lycaenid *Agriades orbitulus *using Pac-Bio and Hi-Fi sequencing. The study is a critical resource in increasing genome sequences for Lepidoptera. The study is well designed, and the methods are thorough. The quality metrics reported suggests that the genome is of sufficiently high quality. Overall, the manuscript is well written and ready for Indexing.

 I have one minor comment to add. I believe that the manuscript will benefit from additional explanation of Merian elements. While the authors provide citation for the relevant paper, I believe a short summary (2-3 lines) about Merian elements and its use in genome assembly would make the paper better.

Are sufficient details of methods and materials provided to allow replication by others?

Yes

Is the rationale for creating the dataset(s) clearly described?

Yes

Are the datasets clearly presented in a useable and accessible format?

Yes

Are the protocols appropriate and is the work technically sound?

Yes

Reviewer Expertise:

Population genetics, bioinformatics, evolutionary genetics, plant-insect interactions, insect-microbe interactions.

I confirm that I have read this submission and believe that I have an appropriate level of expertise to confirm that it is of an acceptable scientific standard.

# Reviewer report: Reviewer response for version 1

**DOI:** 10.21956/wellcomeopenres.27117.r134194

**Published:** 2025-10-24

**Authors:** Melissa Toups, Jaymes Awbrey

**Affiliations:** 1 University of Louisiana at Lafayette, Lafayette, Louisiana, USA

The authors present a chromosome-level genome assembly for the Alpine Blue, *Agriades orbitulus*. The methods are described in detail and are the current best practices for genome assembly. This is a useful genomics resource. I have three comments.

 First, a *de novo* gene annotation would further enhance the utility of this assembly for researchers interested in comparative, population, and functional genomics.

 Second, the Z is identified based on homology to other Lepidopteran assemblies. While this is very likely the Z chromosome, the caveat should be added that this has not been independently verified using coverage analyses or sex-linked polymorphism.  

 Third, Fig. 4 has extensive overlap for the lines for Hap1 and Hap2. Perhaps a dotted or dashed line would allow the reader to view both lines.

Are sufficient details of methods and materials provided to allow replication by others?

Yes

Is the rationale for creating the dataset(s) clearly described?

Yes

Are the datasets clearly presented in a useable and accessible format?

Yes

Are the protocols appropriate and is the work technically sound?

Yes

Reviewer Expertise:

sex-chromosome evolution, comparative genomics, population genomics

We confirm that we have read this submission and believe that we have an appropriate level of expertise to confirm that it is of an acceptable scientific standard.

# Reviewer report: Reviewer response for version 1

**DOI:** 10.21956/wellcomeopenres.27117.r132882

**Published:** 2025-09-30

**Authors:** Han Xu

**Affiliations:** 1 Beijing Forestry University, Beijing, Beijing, China

This study reports the chromosome-level, haplotype-resolved genome assembly of Agriades orbitulus, an important addition to the genomic resources of Lepidoptera. The study design is well-designed, the methods advanced, and the data quality high, with assembly metrics meeting current standards for high-quality genomes. The manuscript is clearly structured, and the data are transparent and open, meeting the indexing requirements for data-based papers. Acceptance for indexing is recommended after minor revisions.

 The manuscript mentions the sample collection site and collaborators but does not clearly state whether relevant collection permits or ethical review approvals were obtained. It is recommended that relevant statements or license numbers be included in the "Methods". And, the title mentioned "DNA barcoding" but the process of molecular identification was not stated.

 The legends of Figure 3 (Merian element distribution) and Figure 4 (k-mer distribution) can be further simplified, and it is recommended that the main conclusions of the figures be briefly stated in the text to enhance readability.

Are sufficient details of methods and materials provided to allow replication by others?

Partly

Is the rationale for creating the dataset(s) clearly described?

Yes

Are the datasets clearly presented in a useable and accessible format?

Yes

Are the protocols appropriate and is the work technically sound?

Partly

Reviewer Expertise:

taxonomy, molecular phylogeny, genomes

I confirm that I have read this submission and believe that I have an appropriate level of expertise to confirm that it is of an acceptable scientific standard.
